# Education and income-based inequality in tooth loss among Brazilian adults: does the place you live make a difference?

**DOI:** 10.1186/s12903-020-01238-9

**Published:** 2020-09-04

**Authors:** Raquel Conceição Ferreira, Maria Inês Barreiros Senna, Lorrany Gabriela Rodrigues, Fernanda Lamounier Campos, Andrea Eleuterio Barros Lima Martins, Ichiro Kawachi

**Affiliations:** 1 Department of Community and Preventive Dentistry, School of Dentistry, Federal University of Minas Gerais, Av. Presidente Antonio Carlos, 6627, Belo Horizonte, Minas Gerais 31270-901 Brazil; 2 Department of Dental Clinic, Surgery and Pathology, School of Dentistry, Federal University of Minas Gerais, Av. Presidente Antonio Carlos, 6627, Belo Horizonte, Minas Gerais 31270-901 Brazil; 3 School of Dentistry, Federal University of Minas Gerais, Av. Presidente Antonio Carlos, 6627, Belo Horizonte, Minas Gerais 31270-901 Brazil; 4 University of Montes Claros, Campus Universitário Professor Darcy Ribeiro, Avenida Rui Braga, S/N, Vila Mauricéia, Montes Claros, Minas Gerais 39401-089 Brazil; 5 Harvard T.H. Chan School of Public Health, Harvard University, 677 Huntington Avenue, Boston, MA 02115 USA

**Keywords:** Tooth loss, Socioeconomic factors, Health status disparities, Adult, Multilevel analysis

## Abstract

**Background:**

Socioeconomic inequalities in tooth loss might be minimized or potentialized by the characteristics of the context where people live. We examined whether there is contextual variation in socioeconomic inequalities in tooth loss across Brazilian municipalities.

**Methods:**

Data from the 2010 National Oral Health Survey of 9633 adults living in 157 Brazilian municipalities were used. The individual socioeconomic indicators were education and household income. At the municipal level, we used the Municipal Human Development Index (HDI) as our contextual indicator of socioeconomic status (low:< 0.699 versus high: > 0.70). The Relative (RII) and Slope (SII) Indexes of Inequality, Relative (RCI), and Absolute (ACI) Concentration Indexes were calculated to compare the magnitude of education and income-based inequalities among municipalities with low versus high HDI. Multilevel Poisson regression models with random intercepts and slopes were developed.

**Results:**

At the individual level, adults with lower education & income reported more tooth loss. The mean number of missing teeth was 9.52 (95% CI: 7.93–11.13) and 6.95 (95% CI: 6.43–7.49) in municipalities with low and high HDI, respectively. Municipalities with high HDI showed higher relative and absolute education-based inequality. For income-based inequalities, higher SII and RCI was observed in municipalities with lower HDI. A significant cross-level interaction indicated that high-education adults reported fewer missing teeth when they lived in municipalities with high HDI compared to adults with the same education level living in low HDI municipalities. For individuals with the lowest education level, there was no difference in the number of teeth between those from municipalities with high and low HDI.

**Conclusions:**

There was a social gradient in tooth loss by education and income. Living in disadvantaged municipalities cannot overcome the risk associated with low schooling. The protective effect of higher education can be reduced when people live in disadvantaged areas.

## Background

Tooth loss is an important oral health problem with consequences for physical and psychosocial health as well as the quality of life [1–3]. It is a good proxy for the cumulative oral health status [4] summarizing the impacts of adverse circumstances throughout the life course of individuals [5].

Despite a significant decline in the prevalence and incidence of severe tooth loss in the past two decades [6], socioeconomic inequalities in this condition persist across the globe, including Brazil [4, 7–9].

The socioeconomic inequalities in tooth loss have been studied considering compositional (individual) and contextual effects based mainly on the pathways linking income inequality to health [10–14]. Metanalysis research with 11 studies showed that adults with lower levels of income presented a greater chance of tooth loss [13]. Regarding the contextual-level, collective evidence has shown associations between high income-based inequality and worse oral health [15]. Compositional explanations suggest that the effect of income inequality is a result of how individual incomes affect oral health. The proposed mechanisms involve the material resources (e.g., the ability to purchase higher quality diets, to afford preventive and regular dental care due to the treatment cost), access to symbolic resources (status and rank within one’s community) and behavioral/cultural explanations, which stress the role of poor health behaviors due to low income (tobacco use, high sugar consumption, infrequent and symptomatic dental visits and poor oral hygiene practices) [14, 16]. Contextual explanations sustain that income inequality has broader psychosocial effects on oral health via stress-induced oral-health-related behaviors [17]. Income inequality also may result from lower social spending on public services and infrastructure, including dental care services and water fluoridation [18].

The income inequality had been a frequently studied contextual social determinant of oral health. However, all dimensions in which social inequality can occur may not be evaluated by this measure. Different levels of education can also explain such disparities, because this socioeconomic indicator may capture the long-term influences of both early life circumstances on adult health, as well as the influence of adult resources on health (for example, through employment status) [19]. Previous studies have shown a consistent independent effect of lower individual education on tooth loss among adults [20–23] and elders [24]. Education equips individuals with knowledge and skills that are useful for the prevention of disease and attitudes toward healthy behavior. Also, higher educational attainment confers greater prestige and status within the community as well as serving as a credential for employment [14].

Beyond the interest in distinguishing the individual (compositional) source of variation from the contextual on socioeconomic inequalities studies, it is crucial to evaluate whether contextual factors affect every income and education group in the same manner [25]. Hence, it is reasonable to assess whether the variations among areas are similar for different income or education groups or whether the variations differ for different education and income groups. This approach is crucial from an equality policy perspective to target interventions to specific groups living in specific areas. Additionally, it takes into account that socioeconomic diversity may minimize or potentialize the adverse oral health consequences of those living in an area with concentrated disadvantage [26].

The influence of contextual determinants on oral health after accounting for individual-level factors (compositional effect) had been extensively shown assuming the fixed effects [18, 20–24, 27–33]. In general, these studies had found higher tooth loss in socially disadvantaged areas independently of individual socioeconomic indicators [22, 24, 32–34]. The evidence also showed attenuation on income inequalities among American adults and Brazilian teenagers by public policies, including dental care services and water fluoridation [18, 35].

A fewer number of studies assessed the interaction between oral health and contextual level factors, and most of them were focused on income inequality [20, 23, 31, 35, 36]. Differential effects of public policies on oral health among individuals with higher education and income had been observed [28, 35]. These results have conducted to discuss if the public policy may both decrease or increase the social gap in oral health, considering the aim, the target population, and the approach of implementation of them (e.g., prioritization of municipalities with better socioeconomic). Can the disadvantage resulting from low education and income be decreased in areas which present more opportunities and conditions for healthy living? Or Do public policies have more effect on groups that are already socially advantaged? The double disadvantage, characterized by concomitantly presenting a worse social situation in the individual and contextual level, has been reported on oral epidemiology [37, 38].

For that, it is crucial to investigate the income and education-based inequality considering the social characteristics of contextual level. A composite indicator of social development can reflect the contextual differences as a result of public policy. The starting point was to estimate the magnitude of income and education-based inequality according to areas with different social development. The aims of this study were, therefore: i) to evaluate income and education-based inequalities in tooth loss comparing Brazilian municipalities with high and low Human Development Index (HDI); ii) to investigate the association between tooth loss and socioeconomic indicators on individual (income and education) and contextual (HDI) levels; iii) to determine if the social environment modifies the relationship between income/education and tooth loss. We hypothesized that tooth loss would be inversely associated with an individual’s household income and education, but that the magnitude of the association would be more pronounced in affluent municipalities (high HDI) than in poor municipalities (low HDI).

## Methods

Data from the present analysis came from the 2010 National Oral Health Survey (SB Brazil 2010) conducted by the Brazilian Ministry of Health in Brazilian urban areas [39] between February and November 2010. The sample was obtained through the random selection of municipalities and census sectors, via multi-stage cluster sampling with probability of selection proportional to population size. Detailed information on the methods is found in other publications [40, 41]. Data for adults aged between 35 and 44 years were used in this study.

Individual interviews using a structured questionnaire was used to obtain demographic and socioeconomic characteristics. Oral health examinations were conducted in people’s homes by calibrated dentists (kappa> 0.65) under natural light following the guidelines of the WHO manual for epidemiological studies [42]. The DMFT (Decayed, Missing, and Filled Teeth) index was used to determine tooth status.

### Outcome variable

The outcome variable was the number of teeth (discrete quantitative variable) that was missing for any reason, determined by the sum of codes 4 and 5 of the DMFT index.

### Exposures

Education and income were used as measures of socioeconomic position at the individual level. Income was measured as total income received by all family members in the month preceding the survey (in seven categories from “R$250.00 or less” to “R$9500.00 or more”). For our analysis, the monthly household income was converted into multiples of the minimum wage, based on the current value at the time of survey (1 minimum wage = R$ 510.00, USD$303.57) and collapsed into four categories: up to 1, 1 to 2.9, 3 to 4.9, 5 or more times the minimum wage. This grouping was defined to distingue mechanisms in which income exerts its effects. Education was asked as the number of years of formal schooling and classified as less than 4 years (insufficient education), 4–7 (incomplete elementary education), 8–10 (completed elementary, but incomplete secondary education), and 11 or more (completed secondary, incomplete university education, or college graduate), according to the formal education system in Brazil.

We used the Municipal Human Development Index (HDI) as an indicator of municipal area socioeconomic status. The Brazilian HDI considers three dimensions: Longevity, Education (access to knowledge, based on average years studied of the population), and Income (living standards and purchasing power of the population according to the Municipal Gross Income per capita) [43]. The HDI was obtained from the 2013 Brazil Atlas of Human Development, which allows a selection based on data extracted from the 2010 demographic. The values of HDI of sampled municipalities ranged from 0.481 to 0.847, and the groups are defined as very low (0–0.499), low (0.500–0.599), medium (0.600–0.699), high (0.700–0.799) and very high (0.800–1.00). According to this classification, the frequency of sampled municipalities was: low and very low (3.93%), medium (7.88), and high and very high (88.19). In this study municipalities were aggregated into low (< 0.699) versus high (> 0.70).

### Covariates

The covariables at individual level were age (adult: 35–39 and 40–45 years old), sex (female, male), skin color (white, black, yellow and brown/Ameridians), time since the last dental visit (< 12 months, between 1 and 2 years, > 3 years, did not visit). Skin color refers to the classification adopted in the demographic census performed in Brazil: whites, blacks, browns, yellows, and Amerindians. In this study, this variable was dichotomized: white versus blacks, browns, yellows, and Amerindians.

At the contextual level, we included the presence of fluoridated water supply (present or absent). The data regarding fluoridation was obtained on the National Basic Sanitation Survey performed by the Brazilian Institute of Geography and Statistics (IBGE) in 2008 [44]. We also included the estimated coverage of the population by primary care oral health services, which corresponds to the mean monthly number of primary care oral health teams for every 3000 individuals to the total population of the municipality in the analyzed year. Higher oral health services coverage indicated higher potential access to basic dental services. The cut off for this variable was 40% that was the goal to be achieved in the biennium 2010/2011 [45]. Data about coverage were obtained from the website of the Department of Information Technology of the Unified Health System (DATASUS).

### Statistical analysis

#### Comparison of income and education-based inequalities between municipalities with high and low HDI

Descriptive analysis was performed to obtain mean tooth loss for each municipality, and the results were shown separately according to the HDI level (high or low). The magnitude of relative and absolute educational and income-based inequalities in the tooth loss was calculated using the Relative Index of Inequality (RII), Slope Index of Inequality (SII) and Relative (RCI) and Absolute Concentration Index (ACI) for municipalities with high and low HDI. RII and SII are summary measures recommended when making comparisons across populations [46]. These indices are regression-based and take the whole socioeconomic distribution into account, rather than only comparing the two most extreme groups. For municipalities with high and low HDI, the population in each education or income category was assigned a modified ridit-score based on the midpoint of the range in the cumulative distribution of the participants in the given category. We used generalized linear models (log-binomial regression), with a logarithmic link function to calculate RIIs (rate ratios) and with an identity link function to calculate SIIs (rate differences) [47]. Both indices were estimated with 95% confidence intervals. The RII can be interpreted as the *rate ratio,* and the SII can be interpreted as the *rate difference* at the bottom and the top of the educational or income hierarchy. If there is no inequality, RII assumes a value of 1.0. The further the value of RII from 1.0, the higher the level of inequality. RII assumes only positive values, with values larger than one indicating a concentration of the indicator among the advantaged and values smaller than one indicating a concentration of the indicator among the disadvantaged. If there is no inequality, SII takes the value of zero. Greater absolute values indicate higher levels of inequality. Positive values indicate higher coverage in the advantaged subgroups, and negative values indicate higher coverage in the disadvantaged subgroups [46]. RCI and ACI were estimated based on the methodology described by Clarke et al. [48]. The RCI provides a measure of the relative differences among education/ income groups. Considering that the RCI was based on morbidity measure (tooth loss), a negative RCI indicates inequality favoring higher income groups. The ACI quantified the absolute differences in health between education and income groups, and this index is not affected by whether it is measured concerning health or morbidity. As RII/SII are mathematically related to RCI/ACI, they will produce the same rank ordering of health inequality between groups but will differ in scale.

#### Association between tooth loss and socioeconomic indicators

Multilevel Poisson regression procedures with unstructured covariances matrix were used to model the two-level structure of individuals (level 1) nested within municipalities (level 2). Multilevel techniques of analyses provide the overall relationship between the individual, compositional factors, and oral health (fixed part), and the variation between areas that cannot be accountable for such factors (random-intercept parameter). Besides, it is possible to assess the variation in certain individual relationships between municipalities (random slope parameters) and the interaction between individual and contextual characteristics (cross-level interactions). A five-step sequential modeling strategy was adopted: i) model 1(empty model): a model without the inclusion of any covariates, in which the variance in tooth loss is inspected between municipalities. A significant random intercept variance indicates the presence of unexplained differences in tooth loss between municipalities. The Wald test evaluated the significance of random intercept, and the Median Rate Ratio measured the heterogeneity among municipalities, according to Austin et al. (2017) [49]. There is no variation between municipalities if the MRR is 1.0, but the higher the MRR, the greater the area-level variation. ii) model 2 (random intercept, fixed effect): considers all the individual-level variables in the fixed part. This model assessed the association between tooth loss and income and education adjusted for covariables at the individual level. The variation between municipalities is allowed for, conditional on the individual, compositional factors. iii) model 3: as model 2, but including the municipalities-level variables. The proportional change in variance (PCV) was calculated according to Merlo et al. (2005) [50], using the following formula: PCV = (variance model 1 – variance model 2)/variance model 1. iv) model 4 (random slope and random intercept model): as model 2, but the model allows both the intercept and slope to be random parameters. Therefore, each municipality has its intercept and slope in which the variability from the overall intercept and slope can be investigated with the addition of individual and municipality variables and their interactions (cross-level interactions). Random slopes for education and income were considered. The comparison of goodness fit between model 2 and model 4 was performed using the LR-test. v) model 5: as model 4, including the municipalities-level variables, in which the cross-level interactions between education/income and HDI were considered. The interaction term represents the change in the slope of education/income on tooth loss across municipalities when HDI changed from low to high. The estimated mean of missing teeth according to individual socioeconomic variables for municipalities with high and low HDI was demonstrated using a graph of the predicted model.

Procedures for complex sample design were used to calculate the proportions of adults according to investigated variables, the age- and sex-adjusted estimates of the outcomes, SII, RII and ACI. Statistical analyses were performed using STATA version 15.0 (StataCorp LP, College Station, Texas, USA).

The Brazilian National Council of Ethics in Research approved the SBBrasil 2010 study, protocol no. 15498, January 7, 2010.

## Results

The sample was 9633 adults, residing in 157 municipalities; 146 individuals in 20 municipalities with a sample of fewer than 10 adults were excluded. The characterization of the sample and mean of missing teeth according to investigated variables are shown in Table 1.

**Table 1:** Proportion of Brazilian adults and mean of tooth loss according to individual and municipalities level variables. Brazil. 2010

	Sample size	Prop. 95% CI^**a**^	Tooth loss^**a**^ Mean (95% CI)
**Individual level variables**			
**Sex**			
Male	3317	36.74	6.59(6.01, 7.16)
Female	6316	63.26	7.81(7.17, 8.44)
**Age**			
35–40	5061	52.39	5.74(5.18, 6.30)
40–45	4572	47.61	9.13(8.42, 9.84)
**Household income (in the minimum wage)**			
up to 1	1388	12.96	10.21(9.13, 11.30)
1 to 2.9	4699	52.90	8.31(7.72, 8.91)
3 to 4.9	1823	20.39	5.60(4.80, 6.41)
> = 5 mw	1480	13.75	3.63(2.92, 4.34)
**Education (in years of schooling)**			
0 to 4	840	10.05	12.09(11.77, 13.41)
5 to 8	2459	29.41	9.34(8.57, 10.11)
9 to 11	3966	37.84	6.70(6.14, 7.27)
> 12	2284	22.70	3.72(3.08, 4.35)
**Self-reported skin color**			
Black + yellow + blown + Ameridians	5558	50.24	7.93(7.35, 8.52)
White	4075	49.76	6.77(6.14, 7.42)
**Time since last dental visit**			
Never used	672	7.32	10.13(8.28, 11.98)
< 1 year	4488	46.69	6.40(5.81, 6.70)
1 to 2 years	2409	25.14	7.39(6.72, 8.06)
> 3 years	1882	20.85	8.39(7.56, 9.11)
**Municipalities level variables**			
**Human Development Index**			
Low + Medium	69	23.72	9.52 (7.93, 11.13)
High	88	76.28	6.95 (6.42, 7.49)
**Coverage of oral health services**			
Below of goal	78	60.82	6.96 (6.40,7.52)
Above of goal	79	39.18	8.31(7.20,9.41)
**Fluoridation of water supply**			
No	52	17.44	9.44(8.48,10.42)
Yes	105	82.56	7.06(6.52,7.59)

95% confidence intervals (CI) in brackets. ^a^Estimates considered weighting and complex sampling design

**Fig. 1:**
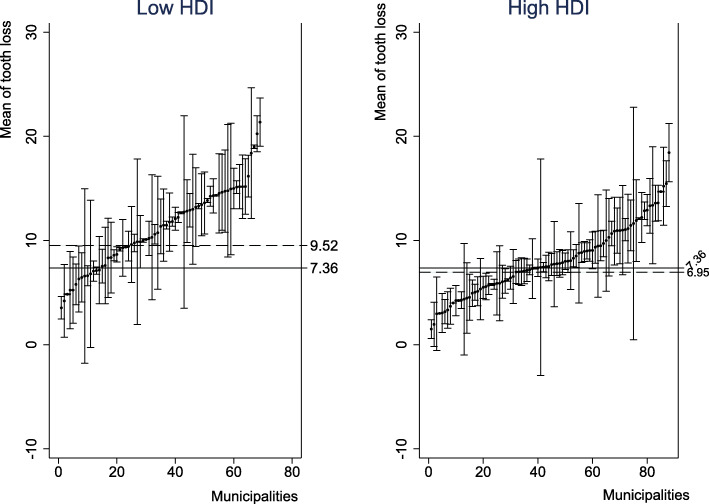
Mean of tooth loss (95% CI) in each Brazilian municipality according to the HDI. HDI – Human Development Index

The mean number of adults per municipalities was 61.36 and ranged from 10 to 496. The mean of HDI was 0.71 (range: 0.481–0.847) and 76.28% presented high HDI. The goal of oral health services coverage was achieved by 39.18 of the municipalities and 82.56 of those had fluoridation of water supply. The mean number of missing teeth among Brazilian adults was 7.36 (SE: 0.26; 95% CI: 6.86–7.87). This value was 9.52 (SE: 0.82; 95% CI: 7.93–11.13) in municipalities with low HDI and 6.95 (SE: 0.27; 95% CI: 6.43–7.49) in those with high HDI (Fig. 1).

The inequalities indexes show a higher number of missing teeth in disadvantaged groups (lowest education and income levels). Higher relative and absolute education-based inequality were observed among municipalities with high HDI. For income-based inequalities, higher SII was observed in municipalities with lower HDI (Table 2).

**Table 2:** Education and income-based inequalities in municipalities with low and high HDI

Education-based inequality			Income-based inequality		
	RII*	RCI*		RII*	RCI*
Brazil	0.27 (0.22;0.34)	−0.19		0.35 (0.28; 0.43)	−0.14
HDI Low	0.41 (0.27; 0.63)	−0.14	Low	0.42 (0.28; 0.62)	−0.12
HDI High	0.27 (0.21; 0.34)	−0.20	High	0.37 (0.29; 0.46)	−0.13
	SII*	ACI*		SII*	ACI*
Brazil	−8.87 (− 10.14; − 7.60)	1.42		−7.63 (− 8.92; − 6.35)	1.06
HDI Low	−8.09 (−11.16; − 5.01)	1.31	Low	− 8.47 (− 11.88; − 5.06)	1.16
HDI High	− 8.54 (−9.91; − 7.17)	1.37	High	−6.91 (− 8.28; − 5.53)	0.95

*SII Slope Index of Inequalities and *RII* Relative Index of Inequalities, *ACI* Absolute Concentration Index, *RCI* Relative Concentration Index

The multilevel crude estimates showed that all individuals variables were significantly associated with tooth loss (Table 3).

**Table 3:** Crude Count Ratio (95% confidence intervals in brackets) of tooth loss according to multilevel models among 34–44 year-olds in Brazil, 2010

	Crude Count Ratio (95% CI)
**Individual level variables**	
**Sex**	
Male	1
Female	1.18 (1.16,1.20)
**Age group**	
35–39 years old	1
40–45 years old	1.59 (1.56,1.61)
**Household income (in minimum wage)**	
up to 1	1
1 to 2.9	0.88 (0.86,0.89)
3 to 4.9	0.68 (0.66,0.70)
> = 5 mw	0.46 (0.45,0.47)
**Education (in years of study)**	
0 to 4	1.00
5 to 8	0.89 (0.87,0.92)
9 to 11	0.65 (0.64,0.67)
> 12	0.41 (0.40,0.42)
**Self-reported skin color**	
White	1
Black + yellow+ brown + Ameridians	1.16 (1.15,1.18)
**Time since last dental visit**	
Never used	1
< 1 year	0.75 (0.73,0.77)
1 to 2 years	0.86 (0.83,0.89)
> 3 years	1.01 (0.98,1.05)
**Municipalities level variables**	
**Municipal Human Development Index**	
Low + Medium	
High	0.71 (0.62,0.81)
**Fluoridation of water supply**	
No	1
Yes	0.77 (0.66,0.89)
**Coverage of public oral health services**	
Below of goal	1
Above of goal	1.18 (1.03,1.36)

Table 4 shows the multilevel models assuming random intercept and fixed effects. The empty model shows that the contextual level variation was significant, suggesting differences in tooth loss among municipalities (LR test: chi2 = 7171.68; Prob > = chibar2 = < 0.0001), and the MRR (1.53) also indicated variation across municipalities. The MRR, as well as the between-municipality variance, come down from 20.09 to 14.17 (− 28.46%) after the inclusion of individual variables (compositional differences) (Model 2) (Table 4). In model 3, assuming fixed effects, a social gradient in tooth loss for income and education was observed. Municipalities with high HDI presented a lower number of tooth loss (Count Ratio: 0.86, 95% CI: 0.76–0.98). The inclusion of municipalities level variables represented an additional 12.7% decrease in variability of tooth loss among municipalities (Table 4).

**Table 4:** Count ratios (95% confidence intervals in brackets) of tooth loss in multilevel models with random intercept and fixed effect between individual and municipalities level variables among 35–44-year old in Brazil, 2010

Parameters	Empty model (Model 1)	Model 2^b^	Model 3^c^
Fixed part	Crude Count Ratio (95% CI)	Adjusted Count Ratio (95% CI)	Adjusted Count Ratio (95% CI)
Individual level variables			
Constant	8.62 (8.02, 9.26)	8.83 (8.22, 9.48)	10.29 (9.09,11.65)
**Education** (in years of study)			
0 to 4		1	1
5 to 8		0.92 (0.90,0.94)	0.92 (0.90,0.94)
9 to 11		0.72 (0.70,0.74)	0.72 (0.70,0.74)
> 12		0.50 (0.49,0.52)	0.50 (0.49,0.52)
**Income** (in minimum wages)			
Up to 1		1	1
1 to 2.9		0.96 (0.94,0.98)	0.96 (0.94,0.98)
3 to 4.9		0.84 (0.82,0.86)	0.85 (0.82,0.86)
> 5		0.65 (0.63,0.67)	0.65 (0.63,0.68)
Municipalities level variables			
**Municipal Human Development Index**			
Low + Medium			1
High			0.86 (0.76,0.98)
Random part			
Area level variance (Random intercept)	20.09 (15.88,25.41)	14.17 (11.15,18.02)	12.37 (9.71,15.76)
PCV^a^		−29.46%	−12.7%
Median Rate Ratio	MRR = 1.53	MRR = 1.43	MRR = 1.39

Results of Multilevel Poisson Regression Model assuming random intercept and fixed effect. Exponentiated coefficients; ^a^PCV: Proportional change in variance. ^b^Model 2: Adjusted for individual-level variables: sex, age group, skin color, and time since the last dental visit. ^c^Model 3: Adjusted for individual and municipalities level variables: sex, age group, skin color, time since the last dental visit, presence of fluoridated water supply, and coverage of public oral health service above the Brazilian goal

Table 5 shows the models whereby the nature of between-municipalities variation was modeled as a function of individual education (random slope). The Likelihood-ratio Test showed that model 4 presented a better fit than model 2 (Likelihood-ratio test: 1204.74, Prob > chi2 = 0.0000), justifying this modeling step. The high-education groups not only have a lower number of tooth loss but also more variance (variance: 0.575 (0.405,0.815) compared to groups with 5–8 years of study (variance: 0.268 (0.271,0.494) and 9–11 years of study (variance: 0.367 (0.272,0.493) (Table 5). The other random slopes were shown as an additional file (Additional file 1). The graph of fitted lines shows how the municipal variation is more pronounced among individuals with higher education (Additional file 2).

**Table 5:** Count ratios (95% confidence intervals in brackets) of tooth loss in multilevel models with random intercept and slope (education) between individual and municipalities level variables among 35–44-year old in Brazil, 2010

Parameters	Random intercept and slope (education)	
	Model 3^a^ Adjusted Count ratio (95% CI)	Model 4^a^ Adjusted Count ratio (95% CI)
Fixed part		
Individual level variables		
Constant	9.18 (8.42,10.00)	9.81 (8.53, 11.28)
**Education** (in years of study)		
0 to 4	1	1
5 to 8	0.82 (0.75,0.91)	0.82 (0.75,0.91)
9 to 11	0.64 (0.57,0.71)	0.64 (0.57,0.71)
> 12	0.44 (0.38,0.50)	0.43 (0.38,0.50)
**Income** (in minimum wages)		
Up to 1	1	1
1 to 2.9	0.94 (0.92,0.96)	0.94 (0.92,0.96)
3 to 4.9	0.83 (0.80,0.85)	0.83 (0.80,0.85)
> 5	0.68 (0.65,0.70)	0.68 (0.65,0.70)
Municipalities level variables		
**Municipal Human Development Index**		
Low + Medium		
High	–	0.95 (0.85,1.22)
**Fluoridation of water supply**		
No		
Yes	–	0.88 (0.78,0.99)
**Coverage of public oral health services**		
Below of goal		
Above of goal	–	1.09 (0.98,1.22)
Median Rate Ratio	1.49	1.48

Exponentiated coefficients; ^a^Model 3 and 4: Adjusted for individual level variables: sex, age group, skin color and time since the last dental visit

Model 5 shows that when the association between education and tooth loss was allowed to vary across municipalities, the main effect of HDI was not significant. The social gradient in tooth loss by individual income and education continue to be observed. Municipalities with fluoridated water showed a lower number of missing teeth (Table 5).

The cross-level interaction between education and HDI was significant, indicating that the association between these two variables is not constant across municipalities, with steeper slopes observed in municipalities with high HDI. There is some evidence that the number of missing teeth is lower for the high-education group when they live in municipalities with high HDI compared with those with the same education level living in municipalities with low HDI. For individuals with the lowest education level, there was no difference in the number of teeth comparing those from municipalities with high versus low HDI. The fixed effect of individual and contextual variables did not change with the inclusion of cross-level interactions (Table 6).

**Table 6:** Rate ratios (95% confidence intervals in brackets) of tooth loss in multilevel models with random intercept and slope (education) and cross-level interactions between HDI and education 35–44 years old in Brazil, 2010

Parameters	.
Fixed part	Adjusted Count Ratio (95% CI)*
Individual level variables	
Constant	9.18 (8.42,10.00)
**Education** (in years of study)	
0 to 4	1
5–8 years of study	0.87 (0.79,0.95)
9–11 years of study	0.78 (0.67,0.89)
> 12 years of study	0.59 (0.48,0.73)
**Income** (in minimum wages)	
Up to 1	
1 to 2.9 minimum wage	0.94 (0.93,0.96)
3 to 4.9 minimum wage	0.83 (0.80,0.85)
> 5 minimum wage	0.67 (0.64,0.69)
Contextual factors (Municipalities level)	
**Municipal Human Development Index**	
Low + Medium	1
High	1.04 (0.90–1.19)
**Fluoridation of water supply**	
No	
Yes	0.85 (0.75–0.96)
**Coverage of public oral health services**	
Below of goal	1
Above of goal	1.17 (0.96–1.19)
Cross-level interactions	
HDI#5–8 years of study	0.99(0.89,1.11)
HDI#9–11 years of study	0.80 (0.66,0.96)
HDI# > 12 years of study	0.67 (0.51,0.88)
Median Rate Ratio (MRR)	1.41

Exponentiated coefficients; *Model was adjusted for individual level variables: sex, age group, skin color and time since the last dental visit

**Fig. 2:**
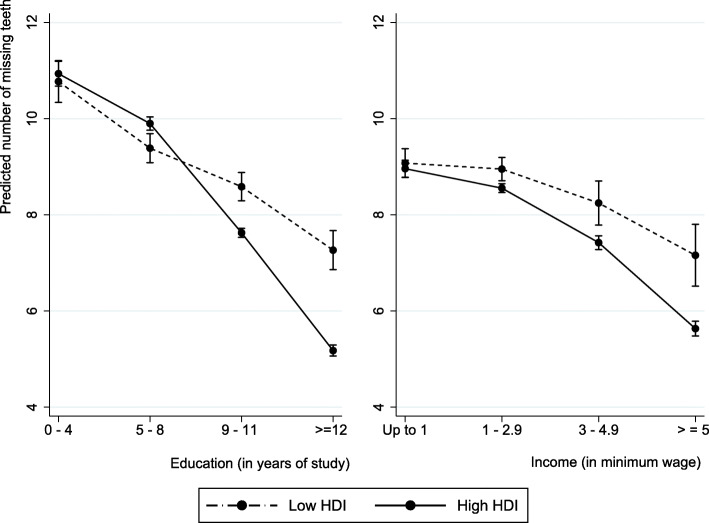
Adjusted predicted effects of education and income on tooth loss in municipalities with low/high HDI

When the variation was modeled as a function of individual income, the model was very similar to obtained with fixed effect (Additional file 3). The graph of fitted lines shows that the municipal variation is similar through income levels (Additional file 4). The cross-level interaction between individual income and HDI was not significant (Fig. 2).

## Discussion

The tooth loss represents an “end state” in oral health, and the present study reaffirmed the persistent concentration of tooth loss among the most disadvantaged individuals. Over and above individual disparities, our findings also demonstrated that the magnitude of educational and income-based inequalities varied between municipalities with high versus low HDI. Our results showed that educational attainment was “less protective” against tooth loss when people live in disadvantaged areas. Conversely, adults with the lowest levels of education and income had greater tooth loss regardless of whether they lived in a municipality with high or low HDI.

Our study replicated the inverse association between socioeconomic status and tooth loss after adjusting for all covariates at the individual and municipality levels [10–12]. Using different outcomes related to tooth loss (< 9 remaining natural teeth; lack of functional dentition/ inadequate dentition: < 21 natural teeth; count of tooth loss or remaining teeth and edentulousness), previous studies have consistently shown more tooth loss among the more disadvantaged groups. Quasi-experimental evidence that worsened economic circumstances (measured by subjective economic deterioration or housing damage due to disaster damage) are associated with tooth loss was provided by a natural experiment following exposure to the 2011 Great East Japan Earthquake and Tsunami [51]. A meta-analysis of 10 cohort studies and two cross-sectional studies found a significant association between low income and tooth loss [13]. Those living in poverty suffer a greater burden of oral diseases, such as dental caries and periodontitis [52, 53], the main causes of tooth loss in adults. They also have a higher prevalence of systemic conditions, such as diabetes, cardiovascular disease, and obesity [54, 55], that are related to tooth loss. Besides these biological explanations, the conceptual mediators of the effect of socioeconomic status on oral health include material deprivation, psychosocial distress, and behavioral factors [14, 17, 19].

Besides, the association between economic constraints and the type of dental treatment delivered has been demonstrated. While subjects in the lower income brackets are more prone to dental extraction, individuals with higher income are more likely to seek periodic routine appointments and conservative dental treatment, resulting in a higher number of retained teeth [56, 57]. In Brazil, the higher tooth loss associated with socioeconomic disadvantage can result from systemic factors related to the organization and delivery of oral health services. The national oral health policy, also known as Smiling Brazil (“Brasil Sorridente”), was implemented only in 2004, ensuring universal coverage [58]. This policy increased the access of adults to oral health services, who historically had significant ongoing unmet needs with only urgent care available in public services [58]. The lack of access to restorative and preventive services in public health services may have contributed to tooth loss, especially among the most disadvantaged individuals. Moreover, previous studies have shown that a change in access to oral health services does not necessarily result in reduced oral health disparities [59]. The explanation was because people take time to change their behaviors, e.g., seeking periodic preventive dental services.

Our results also showed differences in tooth loss inequalities according to municipal HDI, with higher absolute and relative education-based inequality found in municipalities with high HDI and higher absolute income-inequality found in municipalities with low HDI. The high education-based inequality among municipalities with high HDI shows the double disadvantage of adults who share the lowest education and living in places with the worst social indicators [38]. These results suggest that public policies may not reach the persons in the same way, increasing the gap among social groups.

The association between education and tooth loss was not constant across Brazilian municipalities. The significant cross-interaction term reveals that the magnitude of the association between education and tooth loss was lower in municipalities with high HDI. This result suggests that contextual factors may weaken the potential protective effect of schooling at the individual level. Low HDI municipalities in Brazil are smaller cities with lower wealth, higher rates of urban violence, lower sanitation conditions, and urban infrastructure. These contextual aspects may represent fewer opportunities for healthy living for its residents (less access to health services, less availability of diversified healthy food, fewer leisure options, less culture, and social capital). Our result shows that where one lives has an effect on oral health and that this effect must be evaluated considering the characteristics of the people.

On the other hand, the significant cross-level interactions reinforce the hypothesis that public policies can increase the gap among social groups to the extent that it may not reach people in the same way. The cross-interaction was significant after adjusting by oral health policies (coverage of oral health services) and fluoridation of water supply. HDI is an indicator that incorporating a life expectancy, education, and a modified measure of income in a contextual level. However, these indicators may demonstrate the effect of other policies and environmental characteristics that favor access to primary health care in general, sanitation, access to cultural and social opportunities, more social capital, more work and economic security, and more healthy food available. All these contextual factors are potential mediators of the association between HDI and tooth loss and have been pointed out as determinants of oral health [34, 60] in previous studies that had demonstrated the main effect of social-level indicators on tooth loss using multilevel approaches in Brazil, United States, European countries, Australia and Japan [18, 20–24, 27–32].

The analysis performed in this study is advancing, as it may contribute to more targeted policies for the groups with the greatest need, in specific contexts.

The only significant contextual factor we found was the fluoridation of the water supply. Individuals living in municipalities with water fluoridation presented lower tooth loss. The importance of this intervention was previously shown in maintaining functional dentition [21] and tooth retention [23]. Natural experiments applied in oral health context from Brazil showed that adults who accessed fluoridate water < 50% of their lifetime presented a higher mean rate ratio of DMFT index compared with those living > 75% of their life with residential access to fluoridated water. Longer residential lifetime access to fluoridated water was associated with less dental caries, even in the context of multiple exposures to fluoride [61].

We adjusted our models only for key covariates. Similar to the most multilevel studies, the sampling and analytic convenience was the reason for the choice of contextual variables rather than an explicit theory linking area disadvantage and oral health. Therefore, associations among these variables are likely to be underestimated. However, the Brazilian municipalities have to deliver basic education, sanitation, and primary healthcare. Therefore, our chosen contextual factors (HDI, oral health services coverage, and fluoridation of water supply) must fit the area border**.** We used cross-sectional data where the distinction between current and past exposures cannot be made. Similarly, income can change throughout life resulting in upward and downward social mobility.

## Conclusions

There was a social gradient in tooth loss by education and income. Living in disadvantaged municipalities cannot overcome the risk associated with low schooling. The protective effect of higher education can be reduced when people live in disadvantaged areas. This differential effect of education on tooth loss among municipalities with high and low DHI can increase the gap among social groups. The persistent inequality demands for innovative strategies and policies that address the wider social determinants of health, considering the differential effect of public policies among disadvantage and advantage part of society. These policies must protect oral health as a human right through, strengthen intersectoral strategies for poverty reduction, supporting scientific research on the health social determinants, interact communities with public health managers and researchers, support community actions to promote oral health, and eliminate barriers of access to oral health care.

## Supplementary information

**Additional file 1.** Parameters of the random part of the multilevel models with random intercept and slope (education) between individual and municipalities level variables among 35–44-year old in Brazil, 2010.

**Additional file 2.** Municipalities-specific slopes of education on tooth loss.

**Additional file 3.** Count ratios (95% confidence intervals in brackets) of tooth loss in multilevel models with random intercept and slope (income) between individual and municipalities level variables among 35–44-year old in Brazil, 2010.

**Additional file 4.** Municipalities-specific slopes of income on tooth loss.

## Data Availability

The datasets analyzed during the current study are available in the Ministério da Saúde under request. The database request must be send to *Coordenação Geral de Saúde Bucal – Ministério da Saúde/Departamento de Atenção Básic*a, Address: Edifício Premium Torre II – Sala 06 - Setor de Administração Federal Sul – Quadra 2 – Lote 5/6, Brazil. Zip Code: 70070–600. Phone number: + 55 (61) 33159056. Email: cosab@saude.gov.br. The forms to database request are available at the link https://aps.saude.gov.br/ape/brasilsorridente/solicitacaobd.

## Abbreviations

HDI: Municipal Human Development Index
RII: Relative Index of Inequality
SII: Slope Index of Inequality
DMFT: Decayed, Missing, and Filled Teeth
IBGE: Brazilian Institute of Geography and Statistics
DATASUS: Department of Information Technology of the Unified Health System
MRR: Median Rate Ratio
PCV: Proportional change in variance.
