# Supplementary material for: Education and income-based inequality in tooth loss among Brazilian adults: does the place you live make a difference?
Source: BMC Oral Health. 2020 Sep 4;20:246. doi: 10.1186/s12903-020-01238-9 (PMC7650222; doi:10.1186/s12903-020-01238-9)
Supplement: Supplementary file 1 — Additional file 1. Parameters of the random part of the multilevel models with random intercept and slope (education) between individual and municipalities level variables among 35–44-year old in Brazil, 2010. [file 12903_2020_1238_MOESM1_ESM.docx]

Parameters of the random part of the multilevel models with random intercept and slope (education) between individual and municipalities level variables among 35–44-year old in Brazil, 2010.

|  | Random intercept and slope (education) | |
| --- | --- | --- |
| Parameters | Model 3*  Adjusted Count ratio  (95% CI) | Model 4*  Adjusted Count ratio  (95% CI) |
| Random part |  |  |
| Constant | 0.174 (0.127,0.239) | 0.1709 (0.0739,0.2269) |
| Education 5-8 years of study | 0.268 (0.271, 0.494) | 0.2737(0.1967,0.3808) |
| Education 9-11 years of study | 0.367 (0.272,0.493) | 0.3618 (0.2681,0.4883) |
| Education > 12 years of study | 0.575 (0.405,0.815) | 0.5635 (0.4044,0.8135) |
| Covariances (standard error) |  |  |
| Education (5-8), constant | -0.1380 (0.0348) | -0.1402 (0.0308) |
| Education (9-11), constant | -0.1210 (0.0310) | -0.1309(0.0314) |
| Education (> 12), constant | -0.0532 (0.0413) | -0.0652(0.0430) |
