# Supplementary figures and images for: Education and income-based inequality in tooth loss among Brazilian adults: does the place you live make a difference?
Source: BMC Oral Health. 2020 Sep 4;20:246. doi: 10.1186/s12903-020-01238-9 (PMC7650222; doi:10.1186/s12903-020-01238-9)

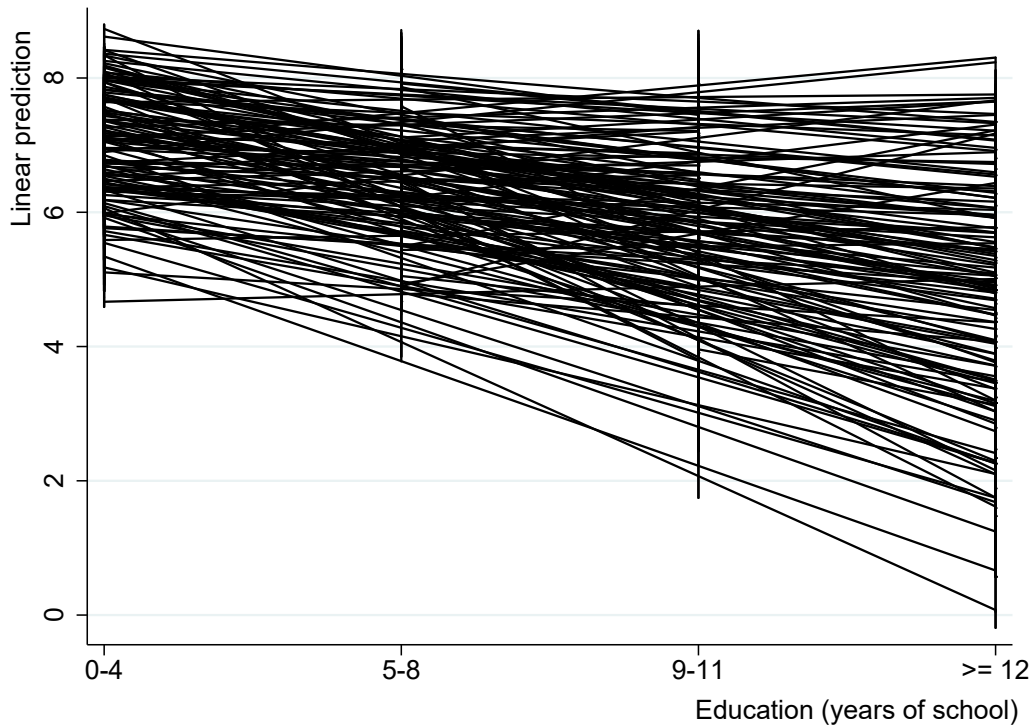

Supplement: Supplementary file 2 — Additional file 2. Municipalities-specific slopes of education on tooth loss. [file 12903_2020_1238_MOESM2_ESM.pdf]

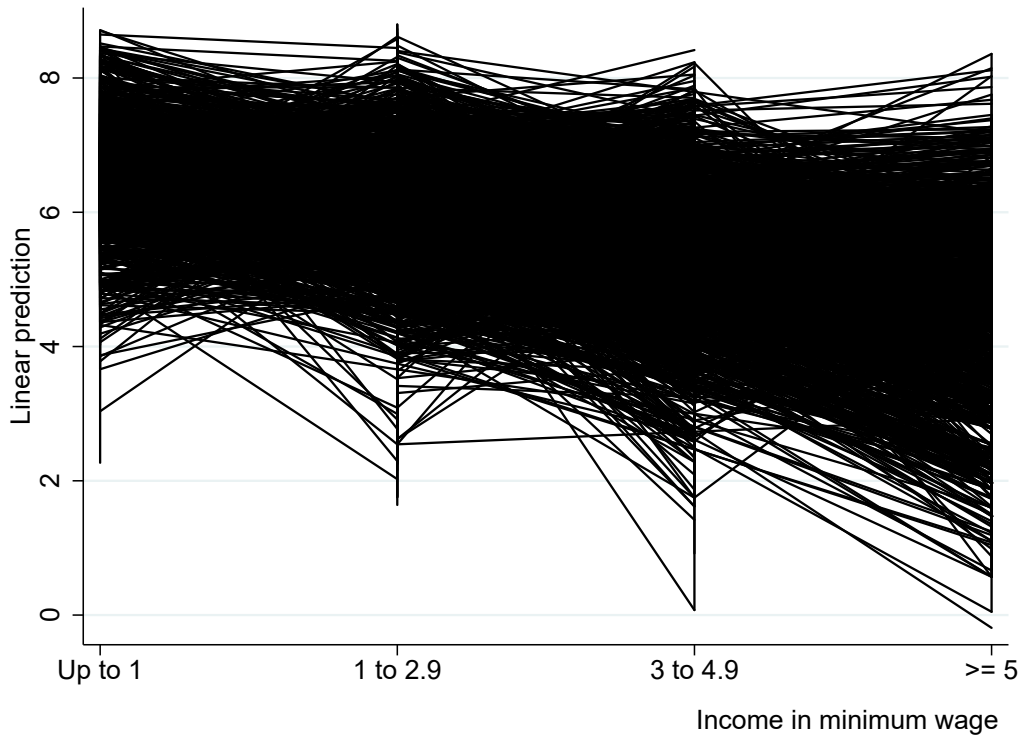

Supplement: Supplementary file 4 — Additional file 4. Municipalities-specific slopes of income on tooth loss. [file 12903_2020_1238_MOESM4_ESM.pdf]
