# Supplementary material for: Education and income-based inequality in tooth loss among Brazilian adults: does the place you live make a difference?
Source: BMC Oral Health. 2020 Sep 4;20:246. doi: 10.1186/s12903-020-01238-9 (PMC7650222; doi:10.1186/s12903-020-01238-9)
Supplement: Supplementary file 3 — Additional file 3. Count ratios (95% confidence intervals in brackets) of tooth loss in multilevel models with random intercept and slope (income) between individual and municipalities level variables among 35–44-year old in Brazil, 2010. [file 12903_2020_1238_MOESM3_ESM.docx]

Count ratios (95% confidence intervals in brackets) of tooth loss in multilevel models with random intercept and slope (income) between individual and municipalities level variables among 35–44-year old in Brazil, 2010.

|  | Random intercept and slope (income) | |
| --- | --- | --- |
| Parameters | Model 4*  Adjusted Count ratio  (95% CI) | Model 5*  Adjusted Count ratio  (95% CI) |
| Fixed part |  |  |
| Individual level variables |  |  |
| Constant | 2.15 (2.06,2.25) |  |
| **Education** (in years of study) |  |  |
| 0 to 4 | 1 | 1 |
| 5 to 8 | 0.91 (0.89,0.93) | 0.92 (0.89, 0.94) |
| 9 to 11 | 0.72 (0.71,0.75) | 0.72 (0.70, 0.74) |
| > 12 | 0.51 (0.50,0.53) | 0.51 (0.50, 0.53) |
| **Income** (in minimum wages) |  |  |
| Up to 1 | 1 | 1 |
| 1 to 2.9 | 0.97 (0.90,1.04) | 0.97 (0.92, 1.02) |
| 3 to 4.9 | 0.74 (0.65,0.83) | 0.85 (0.76, 0.94) |
| > 5 | 0.52 (0.43,0.62) | 0.66 (0.57, 0.76) |
| Municipalities level variables |  |  |
| **Municipal Human Development Index** | | |
| Low + Medium |  | 1 |
| High | - | 0.74 (0.70, 0.90) |
| **Fluoridation of water supply** | | |
| No |  | 1 |
| Yes | - | 0.83 (0.73, 0.94) |
| **Coverage of public oral health services** | | |
| Below of goal |  | 1 |
| Above of goal | - | 0.83 (0.73, 0.95) |
| Random part |  |  |
| Constant | 0.225 (0.169,0.299) | 0.213 (0.167,0.219) |
| Income 1 to 2.9 | 0.173 (0.125, 0.238) | 0.164 (0.135, 0.248) |
| Income 3 to 4.9 | 0.406 (0.285,0.579) | 0.397 (0.274,0.568) |
| Income > 5 | 0.727 (0.494,1.071) | 0.712 (0.473,1.031) |
| Covariances (standard error) |  |  |
| Income (1-2.9), constant | -0.116 (0.0269) | -0.106 (0.0169) |
| Income (3-4.9), constant | -0.099 (0.038) | -0.0879 (0.028) |
| Income (> 5), constant | -0.096 (0.0488) | -0.086 (0.0367) |
